# Reduced graphene oxide-induced crystallization of CuPc interfacial layer for high performance of perovskite photodetectors†

**DOI:** 10.1039/c8ra08864k

**Published:** 2019-01-29

**Authors:** Taoyu Zou, Jianqi Zhang, Shuyi Huang, Chenning Liu, Renzheng Qiu, Xiaozhi Wang, Wei Wu, Hai Wang, Zhixiang Wei, Qing Dai, Chuan Liu, Shengdong Zhang, Hang Zhou

**Affiliations:** School of Electronic and Computer Engineering, Peking University Shenzhen Graduate School Shenzhen 518055 China zhouh81@pkusz.edu.cn; National Center for Nanoscience & Technology Beijing 100190 PR China; Key Lab of Advanced Micro/Nano Electronic Devices & Smart Systems of Zhejiang, College of Information Science and Electronic Engineering, Zhejiang University Hangzhou 310027 China; State Key Laboratory of Optoelectronic Materials and Technologies, School of Electronics and Information Technology, Sun Yat-Sen University Guangzhou 510006 China; London Centre for Nanotechnology, University College London Gower Street London WC1E 6BT UK; Key Laboratory of Yunnan Provincial Higher Education Institutions for Organic Optoelectronic Materials and Devices, Kunming University Kunming 650214 China

## Abstract

Perovskite-based photodetectors have great potential in light-signal conversion; the suppression of the dark current is regarded as one of the main concerns within the academic research communities to achieve a high-performance photodetector. Interfacial engineering in the transport layer is considered as one of the most essential methods for enhancement of perovskite photodetectors. Here, a nanocomposite thin film of tetra-sulfonated copper phthalocyanines and reduced graphene oxide (TS-CuPc/rGO) was investigated as the interfacial layer in perovskite-based photodetectors. Photodetectors with the TS-CuPc/rGO thin film as the interfacial layer exhibited a low dark current density of 2.2 × 10^−8^ A cm^−2^ at bias of −0.1 V as well as high responsivity and detectivity of ∼357 mA W^−1^ and ∼4.2 × 10^12^ jones, respectively; moreover, we observed an ON/OFF ratio of 7.33 × 10^3^ to 520 nm light with an intensity of ∼0.077 mW cm^−2^. Our study revealed that with rGO additives, TS-CuPc molecules were favorable for the formation of an edge-on stacking film with high crystallinity. The rGO-induced crystalline TS-CuPc thin film with lower crystallographic defects effectively reduced the carrier recombination rate at the interfaces, leading to a suppressed dark current and enhanced photocurrent in the photodetector device, when compared to the less crystalline TS-CuPc layer.

## Abstract 2

TS-CuPc/rGO nanocomposite thin films were synthesized and applied as an interfacial layer for high-performance perovskite-based photodiodes.

## Graphical abstract

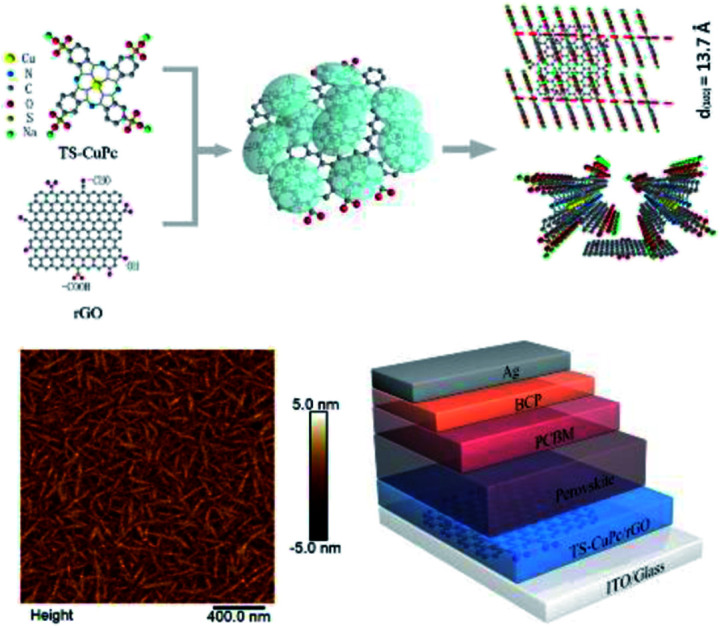

## Introduction

Owing to the remarkable properties including high absorption over the visible spectrum, a long charge carrier lifetime and diffusion length and a tunable band gap,^1–4^ perovskite materials have been regarded as promising materials for next-generation optoelectronic devices such as solar cells,^5–10^ light-emitting devices,^11^ and photodetectors.^12^ Photodetectors based on perovskite materials have a wide range of promising industrial and scientific applications, such as in UV-NIR image sensors^13^ and X-ray detection.^14,15^ Considering device configurations, perovskite photodetectors can be divided into three types: photoconductors, photodiodes, and phototransistors.^16^ Among them, the perovskite photodiode presents the advantages of low dark current, fast response speed, and high detectivity.^2,17,18^ Generally, photodiodes can be operated under a reverse bias or self-powered photovoltaic mode to convert light into an electrical current signal, and detections in extremely weak light, *i.e.*, in the pW range have been reported.^18^ Dark current is an important factor that determines the performance of the photodetector and is closely related to several key figure-of-merit parameters including responsivity (*R*), detectivity (D*), noise equivalent power (NEP), linear dynamic range (LDR) and response speed.^12^ The dark current of a photodiode is significantly influenced by the crystallographic defects in the bulk of the photoactive layer, the transport layer, and the interfaces. The defects in these functioning layers cause charge-carrier recombination and a large leakage current.^19^

Similar to a solar cell, the perovskite photodiode usually adopts p–i–n or n–i–p planar structures.^20^ To suppress the dark current for outstanding performance of the photodetector, high-quality perovskite thin films with large crystallite sizes are usually required to reduce the density of grain boundaries and defects where the trap states are usually located;^19,21,22^ also, the interfacial engineering of electron transporting layers (ETLs) and hole transporting layers (HTLs) is quite critical to boost the performance of photodetectors. The interfacial layer should be designed so as to improve the quality of the perovskite thin film and to passivate its upper and lower surfaces. There is a continuous effort towards optimizing the hole transporting layers and the electron transport layers as well as thin electrode interlayers for perovskite photodetectors. For example, Yang's group investigated the influence of a hole-blocking layer (2,9-dimethyl-4,7-diphenyl-1,10-phenanthroline (BCP) and poly[(9,9-bis(3′-(*N*,*N*-dimethylamino)propyl)-2,7-fluorene)-*alt*-2,7-(9,9-dioctylfluorene)] (PFN)); they demonstrated that with proper device interface design, reduced dark current density of ∼10^−9^ A cm^−2^ and high detectivity of 8 × 10^13^ jones at −100 mV for the perovskite photodetector could be achieved.^23^ Huang's group reported a low-noise perovskite photodetector with the capability of measuring visible light with intensity below 1 pW cm^−2^*via* surface trap passivation by the fullerene layer on the upper perovskite surface.^18^ In another study by Zhu *et al.*, a hybrid NiOx:PbI_2_ nanocomposite was introduced as a hole transport layer for the formation of compact perovskite films and for the passivation on the lower surface of the perovskite; they achieved low dark current density of 2 × 10^−10^ A cm^−2^.^24^

Copper phthalocyanines (CuPc) as classical planar π-conjugated organic molecules with *D*_4h_ symmetry possess the advantages of low cost, thermal and chemical stability, and relatively high mobility, due to which they can be applied as HTLs in perovskite solar cells.^25–28^ Meanwhile, many types of soluble CuPc derivatives, such as copper phthalocyanine-3,4′,4′′,4′′′-tetra-sulfonated acid tetrasodium salt (TS-CuPc) and tetra-propyl-substituted CuPc, have been synthesized for solution-processed devices.^29,30^ However, perovskite photodiodes with metal-phthalocyanine as the transport layer are rarely reported in the literature. This may be because CuPc molecules are more likely to form a thin film with low crystallinity (even an amorphous state at a low deposition or annealing temperature); this may result in interface defects or traps on perovskites, which increase the recombination rate and result in a large dark current. TS-CuPc is a low-cost and water-soluble organic small molecule, and the material synthesis and precursor preparation processes are much easier compared to those of other HTLs. Moreover, both the crystallinity and the stacking orientation of TS-CuPc can be easily controlled by substrate surface treatment at low temperature, rendering it an ideal material for studying the interfacial properties of perovskite photodetectors. Interestingly, previous works have proved that the evaporated metal phthalocyanine molecules can strongly interact with GO or rGO through covalent bonds or noncovalent forces, such as π–π interactions,^31–35^ and the energy level of CuPc/graphene interfaces can be regulated in the interface for semiconductor devices. For example, Kai discovered that the nucleation and orientation of evaporated CuPc molecules on graphene depend on the growth temperature and thickness, with films grown at high temperatures (130 °C) forming face-on orientations throughout the growth process.^36^ Tao reported that with the deposition of CuPc on the graphene, the work function decreased from ∼4.30 eV for pristine graphene to ∼3.90 eV due to the formation of an interfacial dipole at the CuPc/graphene interface.^37^ We also noticed that graphene oxide (GO) or reduced graphene oxide (rGO) has been applied as the HTL or as an additive to modify the HTL in perovskite solar cells due to band alignment matching and high hole conductivity.^38–40^ Inspired by previous studies, here, in an attempt to control the crystallinity of the CuPc organic thin film, we performed a systematic investigation of the effect of the additive rGO on the formation of the solution-processed TS-CuPc thin film; we also studied its impact on the performance of perovskite photodetectors.

Solution-processed TS-CuPc molecules with rGO additives formed a high-crystallinity thin film with an edge-on orientation to the graphene plane, which is beneficial for the reduction of structural defects and extraction of holes. Perovskite photodetectors with TS-CuPc and TS-CuPc/rGO thin films acting as HTLs were demonstrated. Through device interface engineering, the photodetector based on the TS-CuPc/rGO thin film exhibited superior device performance with a low dark current of 2.2 × 10^−8^ A cm^−2^ at −0.1 V, high detectivity of ∼4.2 × 10^12^ jones and a small photoresponse time of 47 ms. These results provide evidence that high-performance photodetectors can be obtained by interfacial engineering with rGO on TS-CuPc thin films.

## Experimental section

### Materials and precursor solution

Unless specified otherwise, all materials and solvents were used as purchased and received. TS-CuPc (85%, Sigma Aldrich) and reduced graphene oxide dispersion (rGO) (Chengdu Organic Chemicals Co. Ltd.) were dissolved in deionized water with solution concentrations of 2 mg ml^−1^, 0.5 wt%, 1 wt%, and 2 wt%. TS-CuPc/rGO solution was obtained by mixing the as-prepared TS-CuPc (1 ml) and rGO (200 μl) solutions. The PEDOT:PSS (Clevios 4083) solution was filtered before use. [6,6]-Phenyl C61 butyric acid methyl ester (PC_61_BM) (Luminescence Technology Corp.) was dissolved in 1,2-dichlorobenzene (Sigma Aldrich) with a concentration of 20 mg ml^−1^. Saturated BCP solution was obtained by dissolving BCP in isopropanol. PbI_2_ was purchased from TCI, and CH_3_NH_3_I and PbCl_2_ were obtained from Xi'an Polymer Light Technology Crop. A perovskite precursor solution was prepared by dissolving PbI_2_ (461 mg), MAI (160 mg) and MACl (3.8 mg) in a mixed solvent of dimethyl formamide (DMF) (640 μl) and dimethyl sulfoxide (DMSO) (80 μl).

### Device fabrication

ITO glass substrates were subsequently cleaned by ultrasonication with deionized water, acetone, and ethanol for 10 min, sequentially, and dried under a nitrogen flow. Then, the substrates were treated with ultraviolet–ozone for 10 min before the deposition of the hole-transport layer. The PEDOT:PSS (Clevios 4083), TS-CuPc and TS-CuPc/rGO solutions were spin-coated onto the cleaned FTO glass at 4000 rpm for 60 s and annealed at 150 °C for 20 min in air. The substrates were then transferred into a glovebox for the deposition of the perovskite layer. The perovskite solution was spin-coated onto the hole transport layer at 4000 rpm for 7 s with anisole as the antisolvent, and the substrates were then baked at 70 °C and 100 °C for 10 min, separately. Afterward, the PC_61_BM solution was spin-coated on top of the perovskite layer at 2000 rpm for 60 s and annealed at 100 °C for 10 min. The BCP solution was spin-coated onto the top of PC_61_BM film at 2000 rpm for 60 s without annealing. Finally, 120 nm Ag contacts were deposited by thermal evaporation under high vacuum (∼5 × 10^−4^ Pa) with a shadow mask for patterned electrodes.

### Material characterization

The surface morphologies of TS-CuPc and TS-CuPc/rGO thin films were observed using atomic force microscopy (AFM, Multimode 8-HR, Bruker, Germany). The work function and Fermi level of the as-prepared TS-CuPc and TS-CuPc/rGO thin films were measured by ultraviolet photoelectron spectroscopy (UPS, Thermo Scientific ESCALAB 250Xi) with He I radiation source (*hν* = 21.22 eV). The UV-vis optical absorption spectra of TS-CuPc and TS-CuPc/rGO thin films were obtained using a Shimadzu UV-2600 UV-visible spectrometer. Raman spectroscopy was carried out using a Raman microscope (inVia Reflex Renishaw) with samples spin-coated on the glass substrates.

### Device characterization

The current density–voltage (*J*–*V*) characteristics and photovoltaic behaviour were measured using a solar simulator (Zolix) under AM 1.5 G (100 mW cm^−2^) and laser sources (520 nm, Shenzhen Optoelectronic Technology Co., Ltd.) by a Keithley 2400 source meter in the glovebox. The photoresponse for different wavelengths of light was recorded by an Agilent B1500A analyzer under monochromatic light produced with a monochromator (Zolix, Omni-λ 3009) in air. The EQE measurements of the devices were carried out in air with the Zolix SCS1011 system. The light intensity was calibrated by using a power meter (Newport, 1936-R). In the measurement process, all devices were illuminated by light from the glass side. The noise power spectrum of the devices was measured by a noise measurement system (Platform Design Automation, Inc., NC300L) from 1 to 10^5^ Hz.

## Results and discussion

**Fig. 1:**
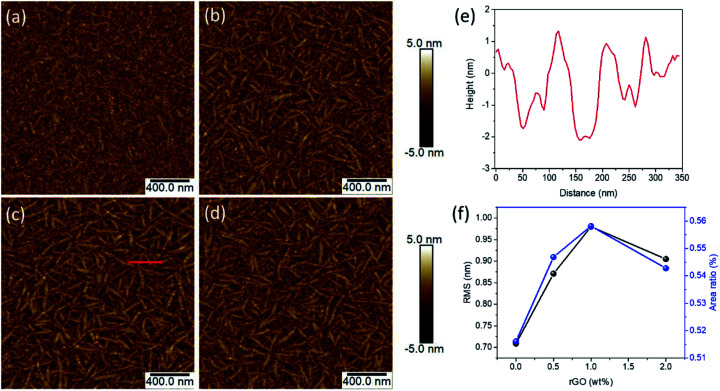
AFM images of (a) bare TS-CuPc thin film, (b) TS-CuPc/rGO (0.5 wt%), (c) TS-CuPc/rGO (1 wt%), (d) TS-CuPc/rGO (2 wt%). (e) The corresponding height profiles from AFM measurements with related position along the red lines in (c). (f) Dependence of the root-mean-square (RMS) and fiber-like crystal ratio on the concentration of doped rGO.

The morphologies of TS-CuPc and TS-CuPc/rGO thin films with different rGO concentrations were scanned by atomic force microscopy (AFM), as shown in Fig. 1a–d. For the thin films without doped rGO (Fig. 1a), the as-prepared TS-CuPc thin films exhibited relative smooth surfaces with small sparse cotton-shaped molecules embedded within the amorphous matrix. In contrast, after the introduction of rGO, the CuPc thin film was transformed into nanorod-aggregated dense surface (Fig. 1b–d), with nanorod-like molecular crystals crossing and overlapping each other. The nanorods exhibited length of ∼200 nm and width of ∼40 nm at 1 wt% rGO (Fig. 1c). Based on an AFM line scan (highlighted in red colour in Fig. 1c), the height of the nanorod crystals (shown in Fig. 1e) was estimated to be ∼2 nm, which was approximately equivalent to the diameter of one CuPc molecule. In addition, it was found that the surface root-mean-square (RMS) roughness also increased from 0.71 nm for films without rGO to above 0.85 nm for films with rGO additives, and the highest value was 0.98 with 1 wt% rGO. The density of the nanorods also followed a similar trend against the concentration of rGO, as presented in Fig. 1f, showing that the coverage ratio of the nanorod crystals also peaked at 1 wt% rGO.

**Fig. 2:**
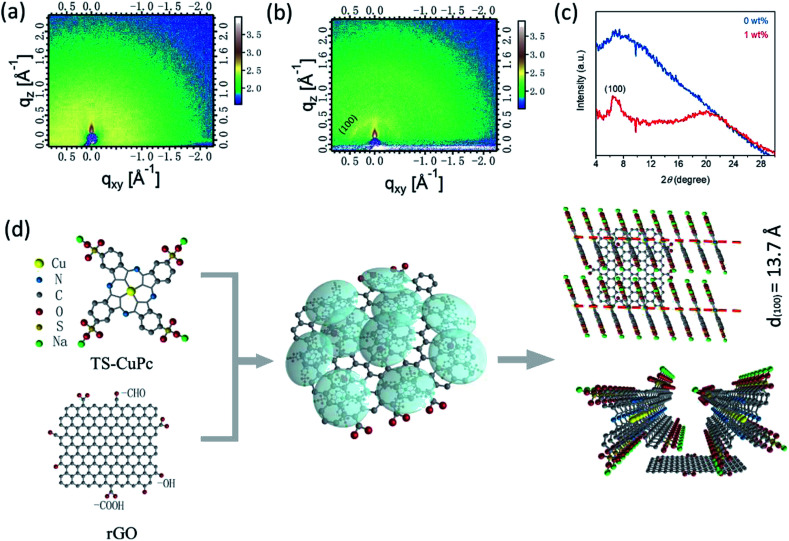
(a and b) 2D GIWAXS patterns of TS-CuPc and TS-CuPc/rGO (1 wt%) and (c) the corresponding integrated intensity patterns for 2D GIWAXS. (d) Schematic representation of the thin film growth mechanism with additive rGO.

To further reveal the crystallinity and molecular stacking orientation of TS-CuPc in the TS-CuPc/rGO thin film, grazing incidence wide-angle X-ray scattering (GIWAXS) patterns for the TS-CuPc and TS-CuPc/rGO films were obtained (Fig. 2a–b). For the TS-CuPc thin film, no clear peaks were observed, but a broad bump centred at ∼6.5° was detected, which implied that the film was majorly amorphous. In contrast, the TS-CuPc/rGO thin film demonstrated a relatively sharp peak at ∼6.43° (13.7 Å), corresponding to the (100) plane reflection of CuPc with interplanar spacing of 13.7 Å. The reduced full-width at half-maximum (FWHM) value (∼1.4°) indicated that larger crystallite sizes were achieved, which agreed well with AFM results. The observed (100) reflection indicated that most portions of the crystallites adopted the edge-on molecular orientation on the substrate. Based on the above GIWAXS results, an rGO-induced crystallite growth mechanism was illustrated (Fig. 2d). Usually, at a low growth temperature, small organic molecules may crystallize into small grains in all directions due to the insufficient energy available for large crystallite growth. The small grains may lead to a large number of defects and traps at the interface, which could degrade the performance of the photodetectors when functioning as the HTL. In an aqueous solution, the added rGO nanosheet can interact with the TS-CuPc molecules *via* noncovalent forces, such as π–π interactions, causing a relatively high concentration of TS-CuPc around the rGO sheets. It is well-known that molecule concentration is one of the most important aspects of crystallization. Therefore, during the annealing step, the aggregation of CuPc molecules around the rGO sheets was advantageous for the formation of large CuPc crystals through π–π stacking. Therefore, the added rGO acted as a bridging agent, facilitating self-assembled TS-CuPc nanorod crystals at a low annealing temperature (150 °C). Thus, we can conclude that the added rGO played an important role in inducing TS-CuPc molecular packing and enhancing the crystallinity of the solution-processed TS-CuPc layer, which may be beneficial for the charge transport and reduction of carrier recombination when functioning as a hole transporting layer.

**Fig. 3:**
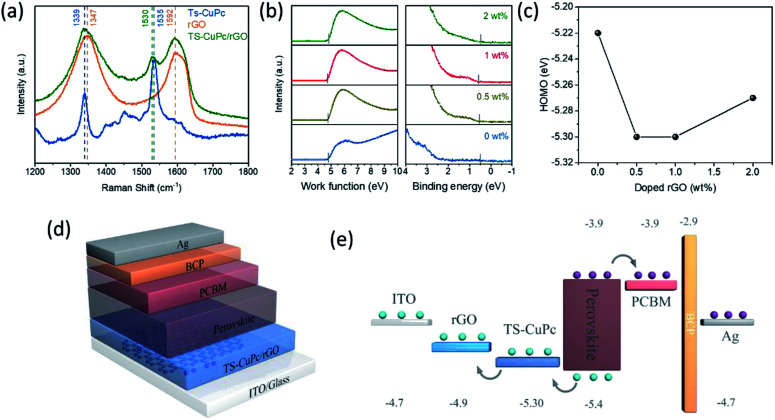
(a) Raman spectra of rGO, TS-CuPc, and TS-CuPc/rGO thin films. (b) Ultraviolet photoelectron spectra (He I: 21.2 eV) of the TS-CuPc thin films with different doped rGO concentrations. (c) Dependence of HOMO level on the concentration of doped rGO. (d and e) Device architecture and the schematic energy structure of photodetectors with TS-CuPc/rGO as the HTL.

Raman spectroscopy is a very powerful technique for studying organic materials. The Raman spectra of rGO, TS-CuPc, and TS-CuPc/rGO thin films were recorded (Fig. 3a). For TS-CuPc thin films, the region between 1300 and 1600 cm^−1^ indicates the fingerprint region for intra-molecular vibrations. Two dominant Raman peaks can be seen at 1339 cm^−1^ and 1535 cm^−1^ due to the B_1g_ mode in-plane vibrations. For rGO, the characteristic peaks are the D-band and G-band located at around 1347 and 1593 cm^−1^, corresponding to the defect mode and the in-plane stretching tangential mode.^31^ The intensity ratio of the D and G bands (*I*_D_/*I*_G_) can be used to evaluate the degree of defects in graphene. In our case, *I*_D_/*I*_G_ obtained from our rGO was calculated to be 1.18, which implied the defective nature of our rGO compound. For the TS-CuPc/rGO composite thin film, typical Raman peaks for TS-CuPc and rGO can still be clearly observed. Meanwhile, *I*_D_/*I*_G_ for TS-CuPc/rGO thin films decreased to 1.07, reflecting the reduction of defect concentration in the structure of rGO; the signature peak of TS-CuPc thin films at 1535 cm^−1^ shifted to 1530 cm^−1^ for the TS-CuPc/rGO composite film, which can be due to the interaction between TS-CuPc and rGO.

As the interfacial electronic structure is of great importance for photoelectric device performance, the electronic structures of TS-CuPc and TS-CuPc/rGO thin films were further revealed by ultraviolet photoelectron spectroscopy (UPS) measurements,^37,41,42^ as shown in Fig. 3b. The optical bandgaps (*E*_g_) calculated using the Tauc plot were 1.69 eV and 1.71 eV for TS-CuPc and rGO (1 wt%)-doped TS-CuPc thin films, respectively (Fig. S1†). The detailed energy levels of TS-CuPc thin film with or without doped rGO are summarized in Table S1.† It was found that with the addition of rGO (1 wt%), the HOMO level of TS-CuPc thin film decreased to 5.30 eV, which was much closer to the valence band of the perovskite; the HOMO level of the TS-CuPc thin film without rGO was 5.22 eV. The lowered HOMO level for the TS-CuPc/rGO film towards the perovskite could lead to a more efficient hole extraction and thus a high photogenerated current density, as confirmed by the *J*–*V* curve.

**Fig. 4:**
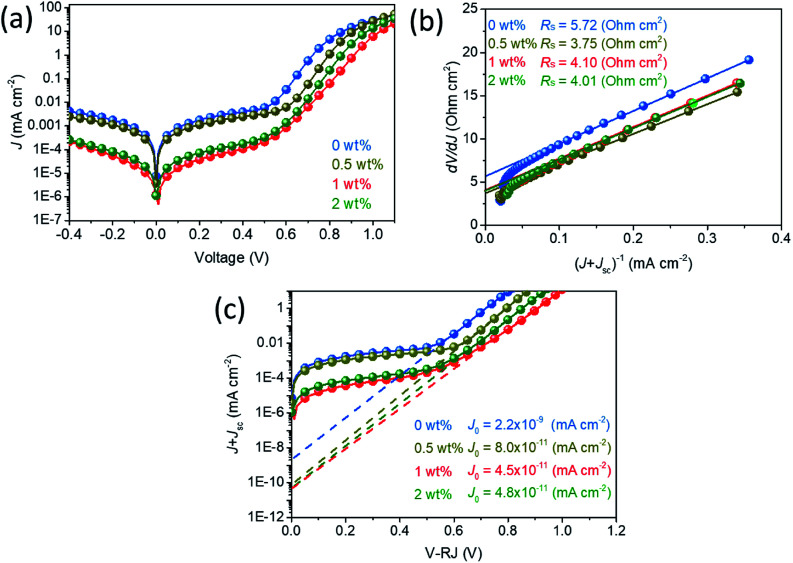
(a) Dark current density–voltage curves of photodetectors with different rGO concentrations. (b) Calculated *R*_s_ of devices by plotting d*V*/d*J versus* (*J* + *J*_SC_)^−1^. (c) Dark saturation current density (*J*_0_) for devices is calculated from the plot of ln(*J* + *J*_SC_) *versus* (*V* + *RJ*).

To test the effectiveness of TS-CuPc/rGO, a perovskite photodiode with TS-CuPc/rGO as the HTL was fabricated; the device structure is shown in Fig. 3d. A schematic of the band structure of the functional layers in the fabricated photodiode is also illustrated in Fig. 3e. Suppressing the dark current density and improving the photocurrent of the device are essential for achieving high-performance perovskite photodetectors. It is worth examining the device features under dark conditions (Fig. 4). As shown in Fig. 4a, the dark current of the device with TS-CuPc/rGO as the HTL is significantly lower than that of the device with only TS-CuPc as the HTL. The dark current density when biased at −0.1 V reduced from 8.4 × 10^−7^ A cm^−2^ for the TS-CuPc-based photodetector to 5.5 × 10^−7^, 2.2 × 10^−8^ and 3.9 × 10^−8^ A cm^−2^ for TS-CuPc/rGO-based photodetectors with the corresponding rGO concentrations of 0.5 wt%, 1 wt%, and 2 wt%. The dark current density of TS-CuPc/rGO (1 wt%)-based perovskite photodetectors was also over one order of magnitude lower than those of rGO- (1 × 10^−6^ A cm^−2^) and PEDOT:PSS-based devices (2.4 × 10^−7^ A cm^−2^), as shown in Fig. S2.† One of the main reasons for the dark current is the recombination current in semiconductors, which can be directly reflected by the dark saturation current density (*J*_0_) extracted from the dark *J*–*V* curve, as shown in Fig. 4b–c. Compared to *J*_0_ for TS-CuPc thin film-based photodetectors (2.2 × 10^−8^ A cm^−2^), *J*_0_ was about 3 orders of magnitude lower for TS-CuPc/rGO thin film-based photodetectors, reaching 4.5 × 10^−11^A cm^−2^ for the device with an rGO concentration of 1 wt%. The improved crystallinity of the TS-CuPc thin film may result in lower defects at the perovskite/TS-CuPc interface, leading to a lower carrier recombination rate in the dark. Moreover, the series resistance decreased from 5.72 to 4.10 ohm cm^2^ in TS-CuPc/rGO thin film-based devices, implying an improved contact between ITO and TS-CuPc assisted by the added rGO.

These results demonstrated that interfacial engineering with TS-CuPc/rGO as the HTL can effectively suppress the dark current of perovskite photodetectors owing to the improved thin film crystallinity of the TS-CuPc thin film and the appropriate passivation of the perovskite film to suppress the activity of the interface defects. Thus, a high-performance perovskite photodetector based on TS-CuPc/rGO thin films as the HTL is expected.

**Fig. 5:**
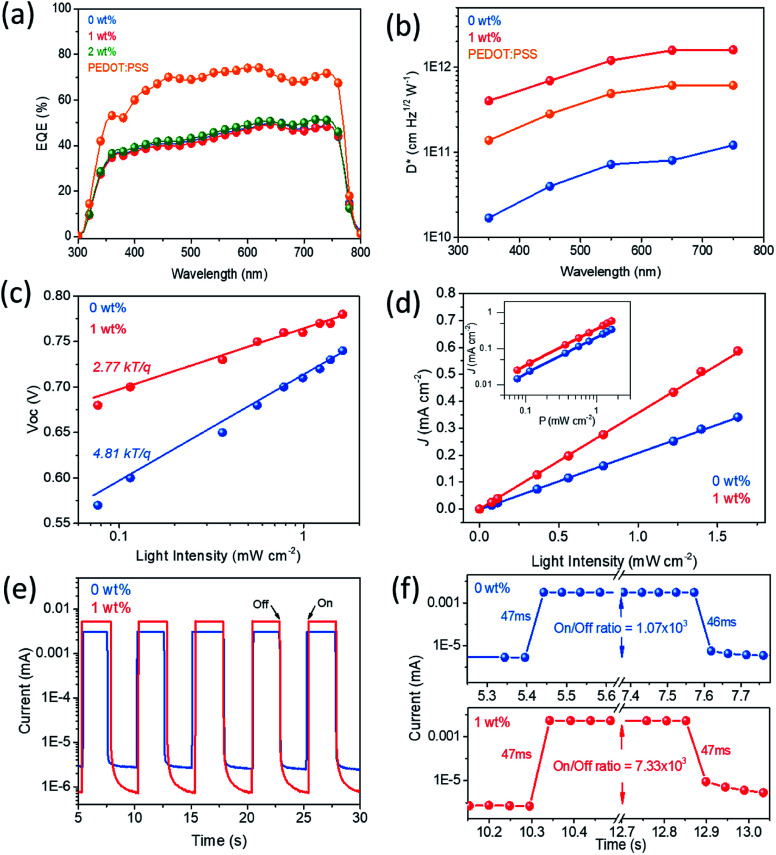
(a) EQE spectra of TS-CuPc thin films with different rGO concentrations and PEDOT:PSS thin film-based photodetectors at 0 V bias. (b) Detectivity of the photodetector-based TS-CuPc, TS-CuPc/rGO (1 wt%), and PEDOT:PSS thin films at different wavelengths at −0.1 V bias with a light intensity of ∼0.070 mW cm^−2^. (c) *V*_OC_*versus* incident light intensity and the corresponding fitted line to the data. (d) Linear dynamic range of devices with TS-CuPc thin films and TS-CuPc/rGO (1 wt%) thin films as the HTL. (e and f) Time response of the photocurrent measured under an incident light of 520 nm at a bias of 0 V.

To further investigate the performance of the perovskite photodetector, the photoresponse characteristics including responsivity, detectivity, and transient response behaviours are presented in Fig. 5. Responsivity (*R*) can be calculated according to the following formula:*R* = EQE*qλ*/*hc* or (*I*_light_ − *I*_dark_)/*P*_0_*A*Here, *q*, *λ*, *h*, *c*, *I*_light_, *I*_dark_, *P*_0_, and *A* are the elementary charge, wavelength, Planck's constant, speed of light, photocurrent, dark current, incident light intensity, and active area, respectively.^12^ Detectivity (D*) is given by (*A*Δ*f*)^1/2^/NEP, where NEP is the noise equivalent power. If the shot noise is the main contribution to the dark current (*I*_dark_), it can be simplified as *R*/(2*qI*_dark_)^1/2^.^43^ It is worth noting that the noise is independent of the frequency from the low frequency of 1 Hz to a high frequency of 10^5^ Hz, indicating that white noise is dominant rather than 1/*f* noise (Fig. S3†).^18^

The external quantum efficiency (EQE) and the corresponding responsivity (*R*) values of photodetectors with or without doped rGO as well as the control device based on PEDOT:PSS thin films are illustrated in Fig. 5a and S4† (between 300 and 800 nm). For TS-CuPc and TS-CuPc/rGO thin film-based devices, the EQE values ranged between 30% and 50% from 350 to 760 nm. The largest *R* value for the TS-CuPc/rGO (2 wt%) thin film-based device was 305 mA W^−1^ at 740 nm, which was slightly higher than that of the TS-CuPc thin film-based device (290 mA W^−1^). In contrast, the control sample with PEDOT:PSS as the HTL showed relatively higher EQE with a peak value of 74% at 620 nm and corresponding *R* of 430 mA W^−1^. The lower EQE obtained from the TS-CuPc device was attributed to the absorption of the TS-CuPc thin film in the UV and visible regions (as shown in Fig. S5† for the UV-vis spectrum). The UV-vis spectrum for metal phthalocyanine originates from molecular orbitals within the aromatic 18-π-electron system and from overlapping orbitals on the central metal.^44–46^ In the near-UV region, the B (or Soret) band, with a peak at 340 nm for the TS-CuPc crystals in this work, can be assigned to the electronic transition between π–π* (b_2u_ to e_g_) orbitals;^47^ in the visible region (the Q band), the peaks at 609 nm and 676 nm may represent the π–π* transition (b_1u_ to e_g_) due to the Davydov splitting.^44^ Nonetheless, despite low EQE values for the TS-CuPc/rGO thin film-based devices, high detectivity (D*) was still achieved with a value of 1.6 × 10^12^ jones at 750 nm, as shown in Fig. 5b. Due to the reduced dark current, the detectivity of the TS-CuPc/rGO (1 wt%) device was over 1 order of magnitude larger than that of the TS-CuPc thin film-based device (1.2 × 10^11^ jones); it was also higher than that of the control sample with PEDOT:PSS as the HTL (6.1 × 10^11^ jones). We further investigated the photoresponse characteristics to monochromatic light (520 nm) within light intensity ranging from ∼0.077 to ∼1.628 mW cm^−2^, as shown in Fig. S6.† Notably, the photosensitivity of the device increased with the doping of rGO into the TS-CuPc thin film. The TS-CuPc/rGO (1 wt%) thin film-based photodetector showed significant improvement in *R* and D*, reaching high values of 357 mA W^−1^ and 4.2 × 10^12^ jones, respectively, compared to the TS-CuPc thin film-based photodetector (*R* and D* values of 207 mA W^−1^ and 4.3 × 10^11^ jones). Since the interface plays a crucial role in enhancing the performance of TS-CuPc/rGO (1 wt%) thin film-based photodetectors, we speculate that the improved thin film crystallinity might help suppress the charge recombination and the adjusted energy level alignments of the transport layer can facilitate charge extraction.

Under dark conditions, with the addition of rGO as the bridging agent to the TS-CuPc molecules, high-quality TS-CuPc thin films were formed in the crystallization process; this significantly decreased the crystallographic defects in the transport layer and the interfaces and consequently suppressed recombination to lower the dark current. Under illumination, since the TS-CuPc/rGO thin films exhibited better crystallinity and a deeper HOMO level compared with the TS-CuPc thin films, holes could be easily extracted from the perovskite thin films. Furthermore, because of the relatively high electrical conductivity and work function of rGO (∼4.9 eV),^39^ the holes in TS-CuPc thin films could transfer to rGO and then to the electrode, resulting in a high photogenerated current. Thus, the introduction of rGO nanosheets into the TS-CuPc thin films not only helped reduce the dark current but also tended to increase the photocurrent of the photodetector, leading to further performance enhancement of the device.

It is important to obtain an understanding of the recombination of the device. There are three contributions to recombination: monomolecular recombination, bimolecular recombination or a combination of monomolecular and bimolecular recombination. Monomolecular recombination, also known as Shockley–Read–Hall (SRH) recombination, is trap-assisted recombination, in which electrons are immobilized in the traps and can subsequently recombine with free holes.^48–50^ On the contrary, a trap-free electron transport feature implies bimolecular recombination or Langevin recombination.^49^ To distinguish among monomolecular recombination, bimolecular recombination and combined recombination, the slope of the curve of *V*_OC_ against light intensity can directly provide us information on recombination.^51^ A slope of *nkT*/*q* (*n* = 1), where *n* is the ideality factor, *k* is the Boltzmann constant, *q* is the elementary charge, and *T* is the temperature (in Kelvin), indicates bimolecular recombination kinetics at the open circuit;^50–52^ due to trap-assisted recombination in the space-charge region, the process transforms to combined recombination with a slope of *nkT*/*q* (1 < *n* < 2) and then to a fully trap-assisted recombination with a slope of *nkT*/*q* (*n* ≥ 2) even at the open circuit.^50,51,53^ The measured *J*–*V* curve under different light intensities is presented in Fig. S7.† Meanwhile, the semi-logarithmic plot of *V*_OC_ depends linearly on the light intensity, as shown in Fig. 5c. The device with TS-CuPc/rGO (1 wt%) has weak *V*_OC_ dependence on the light intensity, with a slope of 2.68 *kT*/*q*, compared to the device without rGO (4.67 *kT*/*q*); this indicates decreased interfacial surface trap sites, suppressed trap-assisted recombination, and therefore enhanced *J*_SC_ because of the improved thin film crystallinity and high conductivity of rGO.

The linear dynamic range (LDR) represents the linearity of the photosensitivity at various light intensities and is defined as 20 × log(*I*_light-max_/*I*_dark_), where *I*_light-max_ is the maximum photocurrent within a linear range; it is calculated in Fig. 5d.^54^ Owing to the low dark current and high light current, the TS-CuPc/rGO (1 wt%) thin film-based device showed LDR as high as 118 dB (at 10 mV), which was much larger than that of the TS-CuPc thin film-based device (LDR, 86 dB) (at 10 mV); this result can also be compared with that of Si photodetectors (120 dB).^54^

Furthermore, the response speed and ON/OFF ratio to the incident light of the devices were tested. Fig. 5e shows the *I*–*t* curves of the photodetectors for an incident monochromatic light of 520 nm with an ON/OFF interval of 5 s for several cycles. Both the photodetectors based on TS-CuPc and TS-CuPc/rGO thin films exhibited a reproducible photocurrent after several ON/OFF light cycles. The photodetectors with or without doped rGO showed a fast photoresponse with rise and fall times of less than 47 ms, as indicated in Fig. 5f; this is the detection limit of our equipment. Please note that devices with TS-CuPc/rGO (1 wt%) thin films as the HTL exhibited lower light-off dark currents and higher light-on photocurrents compared to TS-CuPc thin film-based devices, resulting in a high ON/OFF ratio (7.33 × 10^3^), while that of the TS-CuPc thin film-based device was 1.07 × 10^3^. The performance parameters of the photodetectors based on different HTLs are listed in Table S1† for comparison.

## Conclusions

In conclusion, we demonstrated that the rGO additive could induce TS-CuPc molecules to form high-quality TS-CuPc thin films by low-temperature solution processing. Photodetector devices based on the TS-CuPc thin film with or without doped rGO were then investigated under dark and light illumination conditions. We found that TS-CuPc/rGO thin films as the HTL can significantly suppress the dark current by more than one order of magnitude and increase the photoresponse of the device with high detectivity of ∼10^12^ jones. The high performance of the TS-CuPc/rGO thin film-based photodetector was mainly attributed to the reduced trap-assisted recombination. Furthermore, the TS-CuPc/rGO thin film-based device showed large LDR of 118 dB. This work provides an effective approach for the interfacial engineering of high-performance perovskite-based photodetectors.

## Conflicts of interest

There are no conflicts to declare.

## Supplementary Material

RA-009-C8RA08864K-s001
