# Supplementary material for: Reduced graphene oxide-induced crystallization of CuPc interfacial layer for high performance of perovskite photodetectors
Source: RSC Adv. 2019 Jan 29;9(7):3800–8. doi: 10.1039/c8ra08864k (PMC9060245; doi:10.1039/c8ra08864k)
Supplement: RA-009-C8RA08864K-s001 [file RA-009-C8RA08864K-s001.pdf]

Electronic Supplementary Material (ESI) for RSC Advances.
This journal is © The Royal Society of Chemistry 2019

Electronic Supplementary Information (ESI)

Reduced-Graphene-Oxide Induced Crystallization of CuPc Interfacial Layer for High Performance of Perovskite Photodetector

Taoyu Zou, Jianqi Zhang, Shuyi Huang, Chenning Liu, Renzheng Qiu, Xiaozhi Wang, Wei Wu, Hai Wang, Zhixiang Wei, Qing Dai, Chuan Liu, Shengdong Zhang, Hang Zhou*

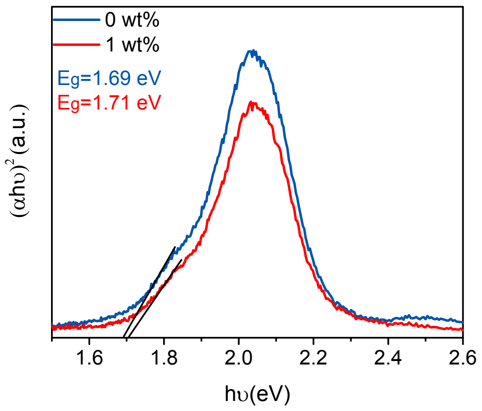

Fig. S1 Bandgaps extracted from Tauc plots with values of 1.69 and 1.71 eV for TS-CuPc and TS-CuPc/rGO (1 wt%), respectively.

Table S1 The detailed energy level of TS-CuPc thin film with or without doped rGO

Doped rGO [wt%]	Work function [eV]	E_f - E_{HOMO} [eV]	HOMO [eV]	LUMO [eV]
0	4.75	0.47	-5.22	-3.53
0.5	4.75	0.55	-5.30	-3.59
1	4.72	0.58	-5.30	-3.59
2	4.80	0.47	-5.27	-3.56

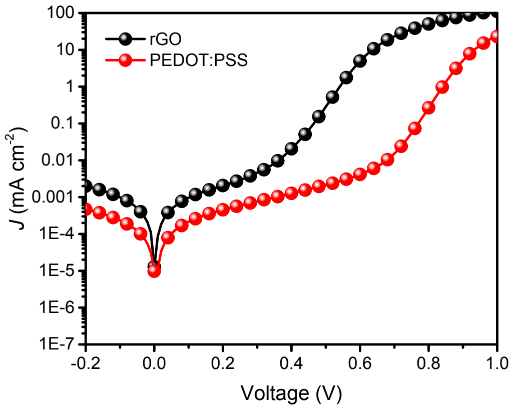

Figure S2. Dark *J-V* curve of perovskite photodetector with PEDOT:PSS and rGO as the HTL

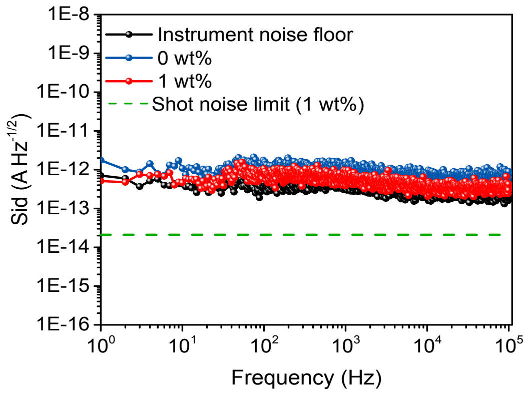

Fig. S3 Measured dark current noise at different frequencies at 0.1 V along with the calculated shot noise limit of TS-CuPc/rGO (1 wt%) thin film-based photodetector.

The noise current is one of the figure-of-merits for photodetector under the dark condition. It can be seen in Figure S2, at a bias voltage of 10 mV. The noise current for TS-CuPc/rGO (1 wt%) thin film-based device is as low as at 5.1×10^{-13} A $Hz^{-1/2}$ at 10 mV and 1 Hz, while that of TS-CuPc thin film-based device is 1.7×10^{-12} A $Hz^{-1/2}$. It is noteworthy that the noise is independent to the frequency from the low frequency of 1 Hz to a high frequency of 10^5 Hz, indicating white noise is dominated rather than 1/*f* noise.[1] However, the equivalent dark current shot noise i_s, which can be expressed as $(2qI_{dark}\Delta f)^{1/2}$ where q is the elementary charge, I_{dark} is the dark current and Δf is the bandwidth,[2] is calculated to be 2.1×10^{-14} A $Hz^{-1/2}$ at 10 mV and 1 Hz for TS-CuPc/rGO (1 wt%) thin film-based photodetector. The difference between calculated and measured noise current may be due to the limitation of the instrument noise floor.

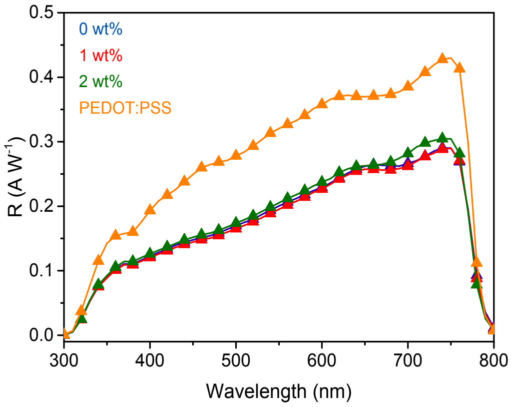

Fig. S4 Responsivity of the photodetector based TS-CuPc thin film with different rGO concentration and PEDOT:PSS thin film as the HTL at different wavelength derived from EQE spectra at 0 V.

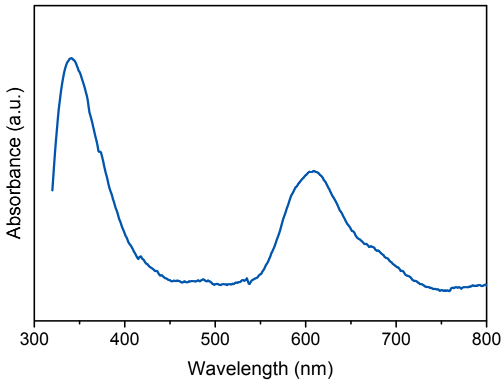

Fig. S5 Absorption spectrum of TS-CuPc/rGO thin film

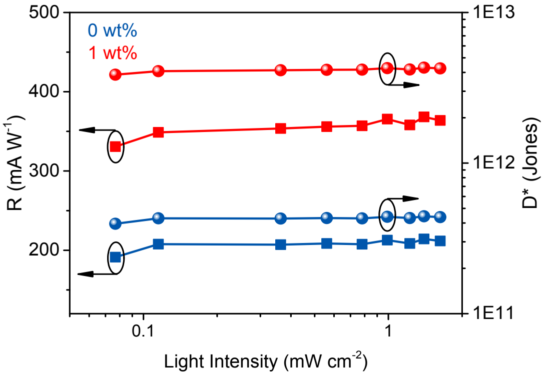

Fig. S6 Responsivity and detectivity of photodetectors based on TS-CuPc thin film and TS-CuPc/rGO (1 wt%) thin film under different incident light intensity at -0.1 V

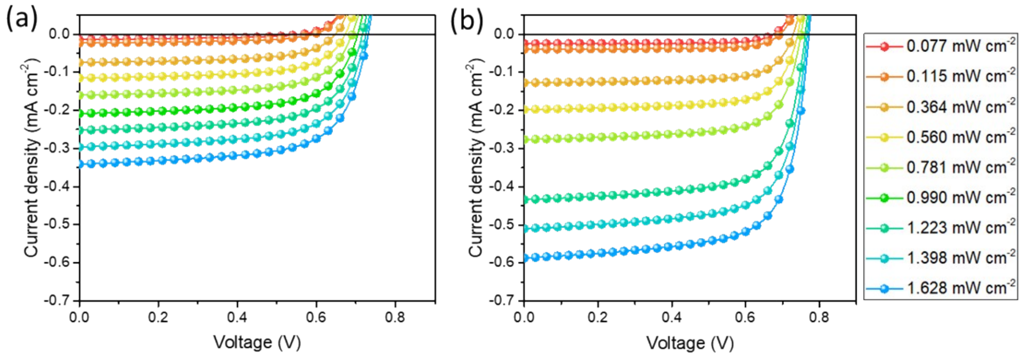

Fig. S7 *J-V* curve of perovskite photodetector under different light intensity with (a) TS-CuPc and (b) TS-CuPc/rGO thin film as the HTL

Table S1. Performances photodetectors based on different hole transporting layers (HTL).

HTL	Device Structure	Dark current/ A/cm^2	Responsivity/ A/W	Detectivity/ Jones	t_{rise}/t_{decay}	Ref.
TS-CuPc:rGO	ITO/TS-CuPc:rGO/Perovskite/PCBM/BCP/Ag	2.2×10^{-8} @ -0.1 V	0.36 @ -0.1 V (520 nm)	4.2×10^{12} @ -0.1 V (520 nm)	< 50 ms	This work
PEDOT:PSS	ITO/ PEDOT:PSS /Perovskite/PCBM/ PFN/Al	~1×10^{-7} @ -1 V	/	8×10^{13} @ -0.1 V (550 nm)	180/160 ns	3
PTAA	ITO/ PTAA /Perovskite/C_{60}/BCP /Cu	1.4×10^{-8} @ -0.3 V	0.47 @ -0.1 V (680 nm)	7.8×10^{12} @ -0.1 V (700 nm)	~1 ns	4
NiO_x:PbI_2	ITO/ NiOx:PbI_2/Perovskite/ C_{60}/BCP /Ag	2×10^{-10} @ -0.2 V	~0.4 (550 nm)	~4×10^{12} (450–750 nm)	58 ns/168 ns	5
OTPD	ITO/ OTPD /Perovskite/PCBM/ C_{60}/BCP/Al	9.1×10^{-9} @ -2 V	~0.21 (white light)	7.4×10^{12} (680 nm)	120 ns	1

References:

1. Y. Fang and J. Huang, Adv. Mater., 2015, 27, 2804-2810.
2. O. Lopez-Sanchez, D. Lembke, M. Kayci, A. Radenovic and A. Kis, Nat. Nanotechnol., 2013, 8, 497-501.
3. L. Dou, Y. M. Yang, J. You, Z. Hong, W. H. Chang, G. Li and Y. Yang, Nat. Commun., 2014, 5, 5404.
4. L. Shen, Y. Fang, D. Wang, Y. Bai, Y. Deng, M. Wang, Y. Lu and J. Huang, Adv. Mater., 2016, 28, 10794-10800.
5. H. L. Zhu, J. Cheng, D. Zhang, C. Liang, C. J. Reckmeier, H. Huang, A. L. Rogach and W. C. H. Choy, ACS Nano, 2016, 10, 6808-6815.
